# The rhythm of horse gaits

**DOI:** 10.1111/nyas.15271

**Published:** 2024-12-28

**Authors:** Lia Laffi, Teresa Raimondi, Carola Ferrante, Eleonora Pagliara, Andrea Bertuglia, Elodie Floriane Briefer, Marco Gamba, Andrea Ravignani

**Affiliations:** ^1^ Department of Human Neurosciences Sapienza University of Rome Rome Italy; ^2^ Department of Life Sciences and Systems Biology University of Turin Turin Italy; ^3^ Fondazione ZOOM Cumiana, Turin Italy; ^4^ Department of Veterinary Sciences University of Turin Grugliasco Italy; ^5^ Behavioural Ecology Group, Section for Ecology and Evolution, Department of Biology University of Copenhagen Copenhagen Denmark; ^6^ Center for Music in the Brain, Department of Clinical Medicine Aarhus University & The Royal Academy of Music Aarhus/Aalborg Aarhus Denmark

**Keywords:** evolution of rhythm, isochrony, locomotion, mammals, rhythmic categories

## Abstract

What makes animal gaits so audibly rhythmic? To answer this question, we recorded the footfall sound of 19 horses and quantified the rhythmic differences in the temporal structure of three natural gaits: walk, trot, and canter. Our analyses show that each gait displays a strikingly specific rhythmic pattern and that all gaits are organized according to small‐integer ratios, those found when adjacent temporal intervals are related by a mathematically simple relationship of integer numbers. Walk and trot exhibit an isochronous structure (1:1)—similar to a ticking clock—while canter is characterized by three small‐integer ratios (1:1, 1:2, 2:1). While walk and trot both show isochrony, trot has a slower tempo and is more precise and accurate, like a metronome. Our results quantitatively discriminate horse gaits based on rhythm, revealing striking commonalities with human music and some animal communicative signals. Gait and vocal rhythmicity share key features, and the former likely predates the latter; we suggest this supports gait‐based hypotheses for the evolution of rhythm. Specifically, the perception of locomotor rhythmicity may have evolved in different species under pressure for predator recognition and mate selection; it may have been later exapted for rhythmic vocal communication.

## Graphical abstract

Most of us are familiar with horse gaits: walk, trot, canter, etc. To our ears, these iconic sounds are intuitively rhythmic. Are they really? This study quantifies the rhythmic structure of these gaits and identifies which characteristics may tell them apart. It also shows that horse gait rhythmicity contains the same patterns as those found across all musical cultures in the world.

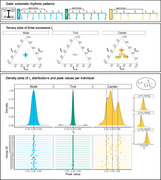

## INTRODUCTION

Temporal regularities, or *rhythm* in its broader definition,^1^ arise in the most diverse natural domains. The morning song of a cuckoo or the chest drumming of a gorilla instinctively resonates in the human perceptual system, raising questions about the adaptive value of a feature that counteracts timing randomness. Animals’ lives are populated by multiple rhythms connected to ecological, physiological, or behavioral processes. Some evolutionary hypotheses link communicative rhythms to gait, suggesting that the perception of rhythmic locomotion patterns might have been a preadaptation for vocal rhythms.^2^ Since locomotion is characterized by a sequence of movements repeated regularly and rhythmically over time,^3^, ^4^ we may hypothesize that the resulting sounds are also rhythmic and that rhythmicity depends on gait. Here, we rhythmically quantify the sound of quadrupedal locomotion to uncover temporal patterns that align with non‐gait rhythms, which have been documented in the recent literature.

How can gait and vocal rhythms be linked? Although the specific circuits involved in rhythm production in nonhuman animals are still partly a mystery,^5^ some evidence suggests that the same substrates involved in locomotion may have represented the ancestral form of rhythms.^3^, ^6^ Previous literature has highlighted that locomotor patterns may play an important role in early brain development, positively affecting human infant rhythm perception.^2^ Moreover, from an ecological point of view, locomotion‐induced sounds produce communicative signals primarily indicating the presence of a moving individual.^7^, ^8^ The ability to perceive and recognize locomotory patterns has considerable adaptive value and may have represented an important preadaptation to developing rhythmic abilities in our own and other species. In humans, the transition to a bipedal gait produced more regular and predictable locomotor sounds; according to some, these sounds may have laid the foundation for proto‐musicality.^9^, ^10^ Locomotor rhythms may have constituted one of the building blocks for the production of rhythmicity at the vocal level.

**FIGURE 1:**
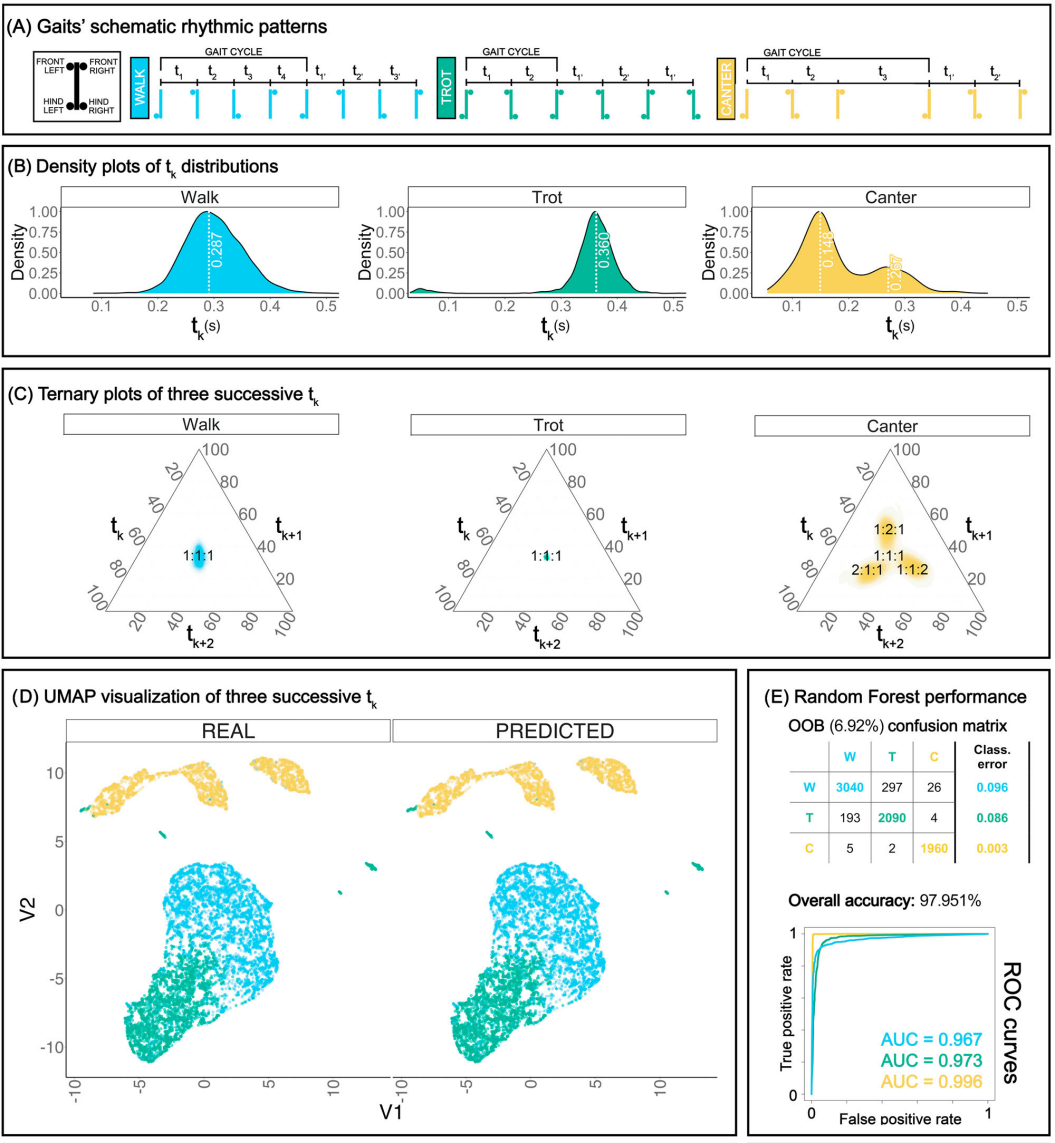
(A) Sequence of footfalls in WALK, TROT, and CANTER; *t*_k_ represents the interval between successive footfalls. (B) Distribution of *t*_k_ values per gait. Peak values are shown on the curve. (C) Ternary plot representation of three adjacent intervals. (D) UMAP visualization of three adjacent intervals, classified based on actual labels (left) versus supervised machine learning (right). (E) Confusion matrix, overall accuracy, and ROC curves describe the high performance of the supervised classification method.

To understand whether locomotor rhythms might be precursors to vocal rhythms in our and other species, it is first necessary to compare temporal patterns between these two different behaviors. Human music and some animal communicative signals share some key rhythmic features: the relationships between adjacent temporal intervals correspond to very simple ratios, that is, small‐integer ratios.^11^, ^12^ Small‐integer ratios provide a powerful metric to analyze any temporal sequence of sounds, allowing comparability of rhythmic patterns across different modalities and species. This study proposes, for the first time, a quantification of the rhythmicity across three distinct gaits. Specifically, here we test for the presence of small‐integer ratios in the sounds produced in three natural horses’ gaits: the WALK, a four‐beat gait; the TROT, a two‐beat gait with two suspension phases; and the CANTER, a three‐beat gait with a single suspension phase^13^ (Figure 1A). We recorded the sounds of the steps of 19 adult and healthy horses and quantified the inter‐onset intervals (*t*_k_) as those intervals between two successive footfalls. First, we used a classification technique to test whether each gait can be discriminated based on the relationships across three adjacent *t*_k_, that is, we tested if a distinctive temporal signature characterizes each gait. Next, we tested whether rhythmic ratios (*r*_k_), that is, the normalized ratios between two adjacent pairs of *t*_k_ (*r*_k_ = *t*_k_/(*t*_k_ + *t*_k+1_)), fall around small‐integer values.^14^ Finally, we quantified how rhythmically precise and accurate the three gaits are.

## MATERIALS AND METHODS

### Study subjects and recordings

We recorded and analyzed the three most common horse gaits: WALK, TROT, and CANTER (Figure 1A). Regarding speed (m/s), WALK is the slowest of the three gaits. It is a four‐beat gait, meaning each hoof strikes the ground separately. The sequence of the hooves hitting the ground is left hind, left front, right hind, right front.^15^ TROT is a two‐beat gait where diagonal pairs of legs move together (left hind and right front together, followed by right hind and left front together^15^). CANTER is a three‐beat gait faster than the trot but slower than the gallop. It has a distinctive sequence of footfalls: one hind leg, the diagonal pair of front and the other hind leg, and finally, the other front leg followed by a suspension phase.^13^, ^16^ We recorded 19 individuals who had been previously declared healthy, based on a thorough clinical examination aimed at detecting signs of lameness. Animals were audio recorded performing WALK, TROT, and CANTER on a firm surface to clearly distinguish the sound of hooves striking the ground. We recorded 1 min of walk, 1 min of trot, and 1 min of canter for each animal, excluding the transition phases between gaits. The horses were ridden by the same rider who performed the recordings by holding an AudioMoth recorder (LabMaker). We recorded shod horses of both sexes (7 females and 12 males), and various breeds, ranging in age from 4 to 28 years and with the height of the withers ranging between 128 and 180 cm.

### Acoustic analysis

We used the software Praat 6.0.56^17^ to analyze the sound recordings. We created a Praat *TextGrid* to annotate the footstep sounds. Particularly, we annotated the sound produced by the hoof striking the ground at the peak of maximum intensity, excluding the transitional phases between gaits. Using a Python script, we then extracted and exported the time series of footsteps from different TextGrids into a .csv datasheet.^18^ The inter‐onset intervals (*t*_k_) were calculated as the time interval between two successive footsteps. The rhythmic ratios (*r*_k_) were calculated by dividing each *t*_k_ by its duration plus the duration of the following interval:^14^ *r*_k_ = *t*_k_/(*t*_k_ + *t*_k+1_). Rhythmic ratios serve to describe local relationships between pairs of adjacent *t*_k_ values.

### Uniform manifold approximation projection and random forest classification

In order to represent the temporal structure of three adjacent *t*_k_ for each gait in a two‐dimensional space while preserving the local structure of the data, we performed a dimensionality reduction via uniform manifold approximation projection (UMAP; *umap* package in R), a nonlinear dimensionality reduction method.^19^ Dimensionality reduction is useful for simplifying the visualization of complex data, such as time series, and facilitates classification algorithms; indeed, high‐dimensional data can be harder to interpret than reduced ones, and sometimes redundant or irrelevant dimensions can obscure meaningful patterns. The obtained coordinates (V1 and V2, serving as the *x* and *y* axes to plot the observations), which recapitulated the relationships existing between three adjacent *t*_k_, were used to perform a supervised classification model. We used the gait type as the classification factor for random forests (*randomForest*; no. of trees = 1000). We extracted 70% of the data to create a Random Forest classifier in the training phase, and the remaining 30% was used to test the model. The model provided an estimated out‐of‐bag (OOB) error, which predicts the expected error of the model on one side and the correctly predicted values on the bootstrapped data on the other side. The model also provided an estimated confusion matrix based on the training performance. The relevance of each variable (V1 and V2) was determined via the *importance* function (*randomForest* R package). We verified that the number of trees was sufficient by plotting the *error ratio* against the number of trees. For the test phase, we used the *predict* function (*randomForest* R package). Receiver operating characteristic (ROC) curves were plotted and the area under curve (AUC) values were extracted for each level of gait with the *performance* function (*ROCR* R package). The percentage of true positives was calculated to quantify the total accuracy of the model, that is, the number of observations in which the true label matched the predicted one. To assess whether the structure of the temporal intervals data was better explained by the gait or the individual, in parallel, we constructed a Random Forest accounting for interindividual differences: in other words, the second model included the same parameters but this time the supervised approach was based on the individual identity instead of gait.

### Comparison of *t*_k_ durations

We compared the median individual *t*_k_ durations of walk and trot using a paired *t*‐test (*t.test* function^20^). We tested the normality of distributions with Shapiro−Wilk tests (*shapiro.test* function^21^).

### Small‐integer ratios test

We followed a methodology commonly used in animal acoustic communication to evaluate the occurrence of small‐integer ratios in the footsteps timing sequences.^12^, ^14^ To test the significance of the peaks of the *r*_k_ distribution, we divided the ratio distribution into on‐integer and off‐integer ratio ranges. The on‐integer ratio ranges were centered around three small‐integer ratios: 1:2, 1:1 (corresponding to isochrony), and 2:1. We centered the off‐integer ratio ranges around 1/3.5 (or 0.285), 1/2.5 (or 0.400), 1‐(1/2.5) (or 0.600), and 1‐(1/3.5) (or 0.710). The on‐integer ratio boundaries were 1/3.25 (or 0.308) and 1/2.75 (or 0.364) for the 1:2 ratio range; 1/2.25 (or 0.444) and 1‐(1/2.25) (or 0.555) for isochrony; and 1‐(1/2.75) (or 0.637) and 1‐(1/3.25) (or 0.693) for the 2:1 category. We counted the occurrences of *r*_k_ within specific off‐ and on‐ratio ranges. We used generalized linear mixed models (GLMMs; package *glmmTMB* ^22^) to test if the numerosity of on‐integer ratios data points was significantly higher than that of off‐integer ratios for each rhythmic category (1:2, 1:1, and 2:1). We ran two GLMMs, one for the WALK and one for TROT, fitting a negative‐binomial distribution. In both models, we treated the count of *r*_k_ as the response variable, defined the specific bin in which the *r*_k_ fell (1:1 on, 1:1 off, 1:2 on, 1:2 off, 2:1 on and 2:1 off) as a fixed factor, and included the horse identity as a random factor. We also entered an offset variable to weight the *r*_k_ numerosity on the width of the bin on the probability density curve. Since each CANTER motion cycle is characterized by three steps and a suspension phase, corresponding to two successive *t*_k_, we tested for small‐integer ratios at the motion cycle level. In other words, the last *t*_k_ corresponds to the suspension phase and is defined by the last beat of a motion cycle and the first of the successive one. For each stride cycle, we thus calculated three *r*_k_: the first one does not include the suspension phase (*r*_k1_ = *t*_1_/(*t*_1_+*t*_2_)), the second one includes the suspension phase (*r*_k2_ = *t*_2_/(*t*_2_+*t*_3_)), and the third one considers the last *t*_k_ of a motion cycle and the first of the successive one (*r*_k3_ = *t*_3_/(*t*_3_+*t*_1’_)). We tested the significance of 1:1, 1:2, and 2:1 rhythmic categories separately with three GLMMs. We used the *r*_k_ as the response variable, and we considered the specific bin (on‐ vs. off‐integer) as a fixed factor and the *r*_k_ type (*r*_k1_, *r*_k2_, *r*_k3_), depending on the specific phase of the motion cycle. The horse identity was used as a random factor. We chose a Poisson distribution for CANTER models and we checked for zero‐inflation and overdispersion (package *performance* ^23^). For all described GLMM models, we conducted a likelihood ratio test by comparing the *full* model, containing all predictors, with a *null* model that included only the random factor and offset to assess its significance. The R *summary* function returned *p*‐values for each predictor and the *emmeans* package, the *p*‐adjustment (Tukey method), and pairwise comparisons.^24^ We checked the normality and homogeneity of residuals by inspecting the residuals’ distribution and the *qqplot* (a function provided by R. Mundry).

### Visualization

**FIGURE 2:**
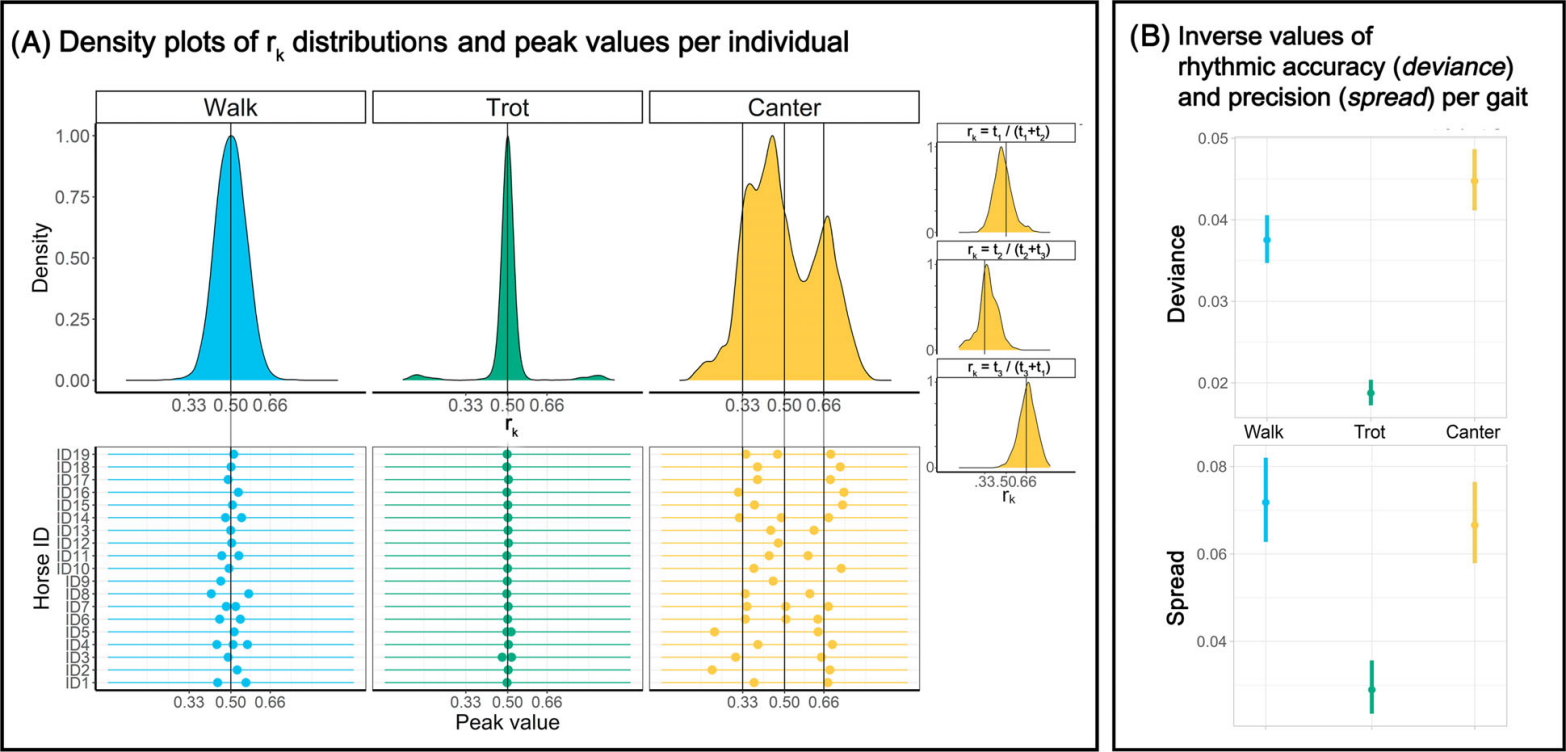
(A) Top: Probability density function of *r*_k_; three additional plots for the canter represent the distribution for each phase of the motion cycle. Bottom: Lollipop plots show the peak values of the ratio density function at the within‐individual level. (B) Accuracy and precision of isochrony for the three gaits.

To visualize the rhythmic structure across different gaits and individuals, we considered four different representations. The density plot of *t*_k_ values (Figure 1B) displays the overall distribution of inter‐onset intervals. The ternary plots (Figure 1C) show the rhythmic structure on the scale of three adjacent *t*_k_ values, representing the relative proportions of the three *t*_k_, ranging from 0 to 100, along the triangle axes. The relationship across three adjacent *t*_k_ has been investigated through dimensional reduction (UMAP) and supervised classification models (Random Forest): the derived two‐dimensional space is represented in Figure 1D, and the performance of machine learning in Figure 1E. The density plot of *r*_k_ (Figure 2A, top) illustrates the distribution of *r*_k_. When the *r*_k_ observations cluster near integer ratio reference values (0.33 for 1:2, 0.5 for 1:1, and 0.66 for 2:1) and this is statistically detectable, this translates into the presence of rhythmic categories following small‐integer ratios. The lollipop plot (Figure 2A, bottom) highlights the local peak values calculated from the *r*_k_ density plot of each individual (*findpeaks* function, *pracma* package).

### Accuracy and precision around isochrony

For all three gaits, we quantified the accuracy and precision of *r*_k_ production for isochrony, the only small‐integer category that was shared across the three gaits. We calculated the accuracy as the deviance from isochrony. Considering all *r*_k_ observations within isochrony boundaries (0.4 < *r*_k_ < 0.6), we calculated the accuracy as the absolute value of the difference between the observed *r*_k_ values and the reference value of isochrony (r_k_ = 0.5). In other words, accuracy quantifies how close the *r*_k_ values are to perfect isochrony. Complementarily, we estimated precision, as the proximity of *r*_k_ values to one another, that is, their spread. For each animal and each gait, the precision was calculated as the difference of the third quartile of *r*_k_ values distribution from the first quartile of the *r*_k_ distribution (i.e., the interquartile range), all within isochrony boundaries (0.4 < *r*_k_ < 0.6). Practically, the wider the interquartile range of *r*_k_ distribution, the higher the spread, the lower the precision. To analyze the differences in precision and accuracy across gaits, we created two separate GLMMs (*glmmTMB* package^22^). The first model used *accuracy* as the response variable, while the second used *precision*. In both models, gait was entered as a fixed factor and horse identity as a random factor. Using the package *fitdistrplus*, we assessed our response variable's most suitable family distribution.^25^ We used a likelihood ratio test to compare the *full* to the *null* model to probe its significance. The R *summary* function provided *p*‐values for each predictor, while the *emmeans* package served for *p*‐adjustment (Tukey method) and pairwise comparisons.^24^ To ensure the assumptions of normality and homogeneity of residuals were met, we examined the distribution of residuals and *qqplots* (a function provided by R. Mundry).

## RESULTS

### Machine learning performance on gait type discrimination

Our model's OOB error was 6.92%, meaning that 93.08% of the new observations were estimated to be correctly classified. The overall accuracy, that is, the ratio between the number of true positives and total observations on training and testing, was 97.95% (Figure 1D). In this performance prediction, WALK's classification error was estimated to be 0.096, TROT's 0.086, and CANTER's only 0.003. The true positive to false positive (AUC; Figure 1E) ratio in the training classification was 0.967 for WALK, 0.973 for TROT, and 0.996 for CANTER.

### Machine learning performance on individual discrimination

To test whether interindividual variability affected the rhythmic structure of three adjacent *t*_k_, we ran an identical machine learning model, supervised on the basis of individuals instead of gait type. This second model showed an estimated OOB error of 81.37%, meaning that 18.63% of the new observations will likely be correctly classified individually. The AUC for the 19 individuals had an average of 0.702 ± 0.073. The overall accuracy of the model was 75.37%.

### Comparison of *t*_k_ durations

We tested if the *t*_k_ values of WALK (M = 0.301, SD = 0.051) and TROT (M = 0.352, SD = 0.062) were different. The values of *t*_k_ were tested with a paired *t*‐test that revealed that TROT displayed significantly longer intervals than WALK (*t* = −10.151, df = 18, *p* < 0.0001).

### Peak significance around small‐integer ratios

The *full* GLMM testing for the small‐integer ratios differed significantly from the *null* model for both WALK and TROT. In particular, both WALK and TROT showed significantly more *r*_k_ falling within the on‐isochrony boundaries than off‐isochrony boundaries (Table 1 and Figure 1F, top). The three *full* GLMMs testing for the small‐integer ratios in the three phases of CANTER also significantly differed from the *null* ones. The first phase (r_k1_ = *t*_1_/(*t*_1_+*t*_2_)) of CANTER showed significantly more *r*_k_ falling within the on‐isochrony boundaries than inside the off‐isochrony boundaries. The second phase (r_k2_ = *t*_2_/(*t*_2_+*t*_3_)) showed a significant peak around 1:2. The third phase (r_k3_ = *t*_3_/(*t*_3_+*t*_1’_)) showed a significant peak around 2:1 (Table 1).

**TABLE 1:** GLMM results for the significance of rhythmic categories spread and deviance.

GLMM models	*Full* versus *null*			Post‐hoc comparisons				
	Chisq	DF	*p*	Contrast	Estimate	SE	Z ratio	*p*
**WALK**	7897.417	5	<0.0001	11off − 11on	−0.834	0.032	−25.742	<0.0001
**TROT**	10463.150	5	<0.0001	11off − 11on	−3.757	0.119	−31.523	<0.0001
**CANTER,** *r*_k1_ = *t*_1_/(*t*_1_+*t*_2_)	567.257	5	<0.0001	11off − 11on	−0.714	0.0724	−9.859	<0.0001
**CANTER,** *r*_k2_ = *t*_2_/(*t*_2_+*t*_3_)	326.356	5	<0.0001	12off − 12on	−0.354	0.090	−4.143	<0.001
**CANTER,** *r*_k3_ = *t*_3_/(*t*_3_+*t*_1’_)	222.817	5	<0.0001	21off − 21on	−0.322	0.087	−3.713	0.003
**Deviance**	1743.258	2	<0.0001	WALK—TROT	0.713	0.019	36.844	<0.0001
				WALK—CANTER	−0.184	0.023	−8.180	<0.0001
				TROT—CANTER	−0.897	0.025	−35.432	<0.0001
**Spread**	47.391	2	<0.0001	WALK—TROT	0.953	0.126	7.572	<0.0001
				WALK—CANTER	0.081	0.099	0.814	0.694
				TROT—CANTER	−0.872	0.127	−6.879	<0.0001

*Note*: Only meaningful *post‐hoc* comparisons are reported.

### Visualization

The distributions of *t*_k_ values show a single peak value at 0.287 s for WALK and 0.360 s for TROT. In the CANTER, two peaks were found at 0.148 and 0.267 s (Figure 1B). The ternary plots show a single cluster around 1:1:1 for both WALK and TROT, and three clusters of points corresponding to 1:1:2, 1:2:1, and 2:1:1 (Figure 1C). The distributions of *r*_k_ show a single peak value at 0.499 for WALK and 0.500 for TROT. In the CANTER, we found three peaks at 0.359, 0.445, and 0.670 (Figure 2A, top). The lollipop plot suggests that WALK produces only one peak (maximum) in the vicinity of 1:1 in 12 out of 19 individuals. TROT produces only one peak in the vicinity of 1:1 in 17 out of 19 individuals; CANTER produces two or three peaks in 17 out of 19 individuals (Figure 2A, bottom).

### Accuracy and precision differences among gaits

Both *full* models testing for differences in deviance (*accuracy*) and spread (*precision*) on the basis of gait differed from the *null* models. Accuracy around isochrony changed among the three gaits, with CANTER showing the highest deviance from isochrony, that is, the lowest accuracy, and TROT showing the lowest values, that is, the highest accuracy. The precision around isochrony was lower in WALK and CANTER than in the TROT, as rhythmic ratios showed higher values of spread (Table 1 and Figure 2B).

## DISCUSSION

WALK and TROT *t*_k_ values showed a unimodal distribution. The CANTER followed a bimodal distribution, suggesting two durational classes of *t*_k_ (Figure 1B). The relationships between three adjacent *t*_k_, displayed by the ternary plots (Figure 1C), were gait specific, with a single cluster around a 1:1:1 ratio for WALK and TROT, and three clusters (1:1:2, 1:2:1, and 2:1:1 ratios) for CANTER. These findings confirm that WALK and TROT are characterized by successive intervals of similar duration.^15^ In the CANTER, a longer interval is associated with the phase of the motion cycle characterized by the suspension phase.^13^

Using a machine‐learning classification approach, we found that the rhythmic patterns of three successive *t*_k_ were significantly different (a) across gaits (Figure 1D,E), with an overall classification accuracy of 97.95%, and (b) across individuals, with an accuracy of 75.37%. Elastic and mechanical constraints arising from the individual morphology can influence the raw durations of *t*_k_, their relationships, and thus the overall locomotor pattern. In other species, gait can be used as a proxy to detect individual identity^26^; in our case, horses are social animals that may gain a decisive adaptive advantage from individual recognition of gait sound. While our models showed an individual‐specific rhythmic pattern, they also clearly evidenced that gait type better explained the differences in rhythmic patterns than interindividuality. In other words, the relationships across three adjacent *t*_k_ were sufficient to differentiate gaits. Gaits show different rhythms, with a potential adaptive role: the sounds of different gaits signal a conspecific's speed,^27^ aiding in collective movement and coordination, which is essential for predation response and interindividual synchronization.^28^, ^29^

Each gait not only had its specific rhythmic signature, but their temporal intervals were also related by small‐integer ratios linking two adjacent *t*_k_ (Table 1). In particular, WALK and TROT showed an isochronous pattern: all intervals had the same duration. By contrast, CANTER showed three different rhythmic ratios resulting from different phases of the motion cycle, corresponding to 1:1, 1:2, and 2:1 small‐integer ratios (Figure 2A). Specifically, the first and second *t*_k_ had equal duration, while the third lasted twice as long. The identified patterns for the three gaits contained slight individual shifts in peak position with respect to the reference value of the small integer but rarely in the number of categories (bottom row of Figure 2A). The small‐integer ratios found in horse gaits are the same as those identified in other species’ vocalization,^30^ suggesting that temporal structures in animal locomotion are remarkably similar to those of other animals’ vocal emissions. Moreover, small‐integer ratios in gaits could explain why humans perceive such patterns as rhythmic; some rhythmic features transcend musicality and animal communicative signals.

The analysis of ratios revealed a clear difference between CANTER, with three small‐integer rhythmic categories, and WALK and TROT, both displaying an isochronous sequence. But which rhythmic features distinguish WALK from TROT? To assess the regularity of rhythms and its role in gait discrimination, we quantified the rhythmic accuracy and precision: we found that TROT showed the highest accuracy and precision values around isochrony. One reason for that could lie in that TROT shows a simpler spatial arrangement and symmetrical limb coordination pattern, with mirrored alternation of two limbs, giving fewer opportunities for symmetry breaking compared to the WALK (four‐beat) and CANTER (three‐beat with a suspension phase).^31^ If we consider TROT to have the simplest locomotor pattern and exhibiting the highest dynamical stability, corresponding to the easiest small‐integer ratio (1:1), then the lower interval variability stands to reason.^32^ Also, each gait can be performed in a certain speed range, but it has been shown that minimal interstride variability can be reached at a specific optimal speed.^27^ Indeed, speed might affect the accuracy and precision of gaits. Since Figure 1B suggests that the animals showed less variability in speed at TROT than WALK, this would explain TROT's higher rhythmic regularity. However, further research is required to better explore the connection between rhythmic regularity and locomotor speed. Our results suggest that the discrimination between WALK and TROT, which exhibit an isochronous pattern, can be based on the higher regularity of TROT and the faster tempo of WALK.

## CONCLUSION

This study quantified the rhythmic characteristics of horse gaits. Each gait has its own rhythmic signature, potentially allowing gait recognition. Overall, the sound of horse locomotion shows high periodicity, supporting evolutionary hypotheses: in species needing efficient endurance locomotion, maintaining a regular pace is energetically adaptive and has probably been reinforced by proximate rewards throughout evolution.^9^ Crucially, we show that horse locomotion shares a crucial rhythmic property with animal vocalizations and human music, that is, small‐integer ratios. Our findings reveal that locomotor patterns exhibit the same rhythmic key features found in communicative signals of other species, supporting the gait‐based hypothesis of rhythm, which posits that the ability to keep a regular gait may predate the evolution of complex rhythmic behaviors.^2^ In species showing rhythmic vocalizations, this might be the result of a bidirectional coupling between locomotion and respiration, as a groundwork for a link between respiration and phonation^33^: since phonation is a highly expensive process, the energetic advantage of coupling movement and breathing could be reflected in shaping the vocal signal on the same time grid. The specific neural circuits involved in rhythm perception and production across species and their sensitivity to early sensory experience have yet to be elucidated.^5^ Still, the functional and anatomical connections between spinal‐ and supra‐spinal regions in locomotion^3^ suggest that these rhythms may have represented the ancestral form of rhythms in taxa that also show rhythmic communicative displays, as already proposed for humans.^9^

## AUTHOR CONTRIBUTIONS

Conceptualization: L.L., M.G., and A.R. Methodology: L.L., T.R., M.G., and A.R. Data collection: L.L. and C.F. Formal analyses: L.L. and T.R. Writing—original draft: L.L., T.R., and A.R. Writing—review and editing: all authors. Visualization: L.L. and T.R. Supervision: M.G. and A.R.

## COMPETING INTERESTS

All authors declare no competing interests.

### PEER REVIEW

The peer review history for this article is available at: https://publons.com/publon/10.1111/nyas.15271

## Data Availability

The datasets used for the current study are available from the corresponding authors upon reasonable request.
